# Role of aspartyl-(asparaginyl)-β-hydroxylase mediated notch signaling in cerebellar development and function

**DOI:** 10.1186/1744-9081-6-68

**Published:** 2010-11-04

**Authors:** Elizabeth Silbermann, Peter Moskal, Nathaniel Bowling, Ming Tong, Suzanne M de la Monte

**Affiliations:** 1 Warren Alpert Medical School of Brown University, Providence, RI, USA; 2 Brown University, Providence, RI, USA; 3 Department of Medicine, Rhode Island Hospital, Providence, RI, USA; 4 Departments of Pathology, Neuropathology, and Neurology, Rhode Island Hospital, Providence, RI, USA

## Abstract

**Background:**

Aspartyl-(Asparaginyl)-β-Hydroxylase (AAH) is a hydroxylating enzyme that promotes cell motility by enhancing Notch-Jagged-HES-1 signaling. Ethanol impaired cerebellar neuron migration during development is associated with reduced expression of AAH.

**Methods:**

To further characterize the role of AAH in relation to cerebellar development, structure, and function, we utilized an in vivo model of early postnatal (P2) intracerebro-ventricular gene delivery to silence AAH with small interfering RNA (siAAH), or over-express it with recombinant plasmid DNA (pAAH). On P20, we assessed cerebellar motor function by rotarod testing. Cerebella harvested on P21 were used to measure AAH, genes/proteins that mediate AAH's downstream signaling, i.e. Notch-1, Jagged-1, and HES-1, and immunoreactivity corresponding to neuronal and glial elements.

**Results:**

The findings demonstrated that: 1) siAAH transfection impaired motor performance and blunted cerebellar foliation, and decreased expression of neuronal and glial specific genes; 2) pAAH transfection enhanced motor performance and increased expression of neuronal and glial cytoskeletal proteins; and 3) alterations in AAH expression produced similar shifts in Notch-1, Jagged-1, and HES-1 protein or gene expression.

**Conclusions:**

The results support our hypothesis that AAH is an important mediator of cerebellar development and function, and link AAH expression to Notch signaling pathways in the developing brain.

## Background

Aspartyl-(asparaginyl)-β-hydroxylase (AAH) is an ~86 kD Type 2 transmembrane protein and member of the α-ketoglutarate-dependent dioxygenase family that includes prolyl-3, prolyl-4, and lysyl hydroxylases [1-3]. AAH's carboxyl region can be proteolytically cleaved to generate ~52 kD or ~56 kD catalytically active fragments [1,3,4]. Site-directed mutagenesis studies demonstrated that the ^675^His residue present in the C-terminal fragment is essential for catalytic activity [1,5]. AAH catalyzes post-translational hydroxylation of β carbons of specific aspartate and asparagine residues in epidermal growth factor (EGF)-like domains [6] of proteins such as Notch and Jagged [5,7], which have known roles in cell growth, differentiation, and neuronal migration during development [8,9], and in extracellular matrix molecules, such as tenascin [2], which mediate adhesion, motility, and cell process extension [10-12]. Correspondingly, previous studies showed that Jagged, the ligand for Notch [13,14], is indeed a substrate for AAH hydroxylation [7], and that AAH is capable of physically interacting with both Notch and Jagged [15]. Moreover, over-expression of AAH results in increased nuclear translocation and accumulation of Notch, and activation of Notch's downstream target genes, including Hairy and Enhancer of Split 1 (HES-1) [15].

A direct role for AAH in cell motility and invasion was demonstrated by the findings that: 1) over-expression of AAH by transfection with recombinant plasmid DNA increases cell motility; 2) inhibition of AAH via gene silencing with small interfering (si) RNA duplexes reduces cell motility; and 3) inhibition of signaling pathways required for AAH expression and function impairs cell motility [15-21]. The AAH gene is regulated by insulin and insulin-like growth factor (IGF) signaling through insulin receptor substrate (IRS)-dependent pathways that activate Erk MAPK and phosphatidylinositol-3-kinase (PI3 kinase)-Akt [15,17,19]. However, AAH is also regulated by post-translational mechanisms, since chemical inhibition of glycogen synthase kinase 3β (GSK-3β) by LiCl or transfection with si-GSK-3β [16,19] increased AAH protein without altering its mRNA levels, and over-expression of catalytically active GSK-3β increased AAH phosphorylation and reduced AAH protein expression [16].

Previous studies demonstrated that ethanol inhibits insulin and IGF signaling in immature neuronal cells [22-26], and that chronic in utero exposure to ethanol causes fetal alcohol spectrum disorders (FASD). FASD is associated with impaired cerebellar development including hypoplasia, disordered neuronal migration, insulin and IGF resistance, and reduced AAH expression [18,24-27]. Ethanol's inhibitory effects on AAH are mediated at transcription and post-translation levels [18]. Since insulin and IGF signaling pathways mediate cerebellar growth and development [28], and AAH is a downstream target of insulin and IGF stimulation [15,19], we hypothesize that in FASD, ethanol impaired cerebellar development is mediated, in part, by inhibition of AAH expression and/or function. Herein, we used in vivo models to determine if inhibition of AAH is sufficient to cause some of the functional and neuro-developmental abnormalities observed in FASD. The strategy used was to transfect immature brains with siRNA targeting AAH, or recombinant plasmid carrying the full length AAH cDNA, and examine the long-term consequences in terms of function, structure, and gene expression in the brain. We focused our investigations on the cerebellum because this structure: 1) develops mainly in the early postnatal period; 2) is a primary target of ethanol-mediated neurotoxicity; and 3) exhibits impaired AAH expression in experimental models of FASD [18].

## Methods

### Gene delivery model

Two-day-old (P2) Long Evans rat pups were given a single intracerebroventricular injection of small interfering RNA duplexes (siRNA) that targeted AAH (siAAH) [ASPH NM_001009716] or no specific sequences (scrambled; siScr) [NM D-00121001-20], or recombinant plasmid DNA containing the complete coding sequence of human AAH (pAAH), or Green fluorescent protein (pGFP). The cDNAs were ligated into the pcDNA3.1 vector (Invitrogen, Carlsbad, CA) in which gene expression was under the control of a CMV promoter. Supercoiled plasmid DNA was purified using endotoxin-free columns (Qiagen Inc., Valencia, CA). For each animal, 10 μg of recombinant plasmid DNA or 0.4 nmol siRNA were complexed with 10 μl of Dharmafect reagent (Dharmacon, Inc., Chicago, IL), and injected into the right lateral ventricle using a Hamilton syringe with a 26-gauge needle as previously described [29,30]. All animals survived the procedure, and there were no consequential aberrant behaviors or adverse effects such as failure to thrive, poor grooming, reduced physical activity, or weight loss. The rats were subjected to rotarod testing on P20, and sacrificed on P21 (N = 8 per group). However, several rats were sacrificed on P35 for longer observation (N = 6 per group). Cerebella were divided in the mid-sagittal plane. One half was fixed in Histochoice (Amresco, Solon, OH) and embedded in paraffin. Histological sections were stained with Luxol fast blue, hematoxylin and eosin (LHE) to detect morphological abnormalities. The other half was snap-frozen in a dry ice/methanol bath and stored at -80°C for later mRNA and protein studies. Our experimental protocol was approved by the Institutional Animal Care and Use Committee at Lifespan-Rhode Island Hospital, and it conforms to the guidelines set by the National Institutes of Health.

### Rotarod testing

We used rotarod testing to assess long-term effects on motor function [31] resulting from the siAAH or pAAH treatments. On P19, rats were trained to remain balanced on the rotating Rotamex-5 apparatus (Columbus Instruments) at 1-5 rpm. On P20, rats (N = 8-10 per group) were administered 10 trials at incremental speeds up to 10 rpm, with 10 minutes rest between each trial. The latency to fall was automatically detected and recorded with photocells placed over the rod. However, trials were stopped after 30 seconds to avoid exercise fatigue. Data from trials 1-3 (2-5 rpm), 4-7 (5-7 rpm), and 8-10 (8-10 rpm) were culled and analyzed using the Mann-Whitney test.

### Quantitative reverse transcriptase polymerase chain reaction (qRT-PCR) analysis

We used qRT-PCR to measure mRNA expression as previously described [15,30,32]. In brief, cerebella were homogenized in Qiazol reagent (Qiagen Inc., Valencia, CA), and total RNA was isolated using the EZ1 RNA universal tissue kit and the BIO Robot EZ1 (Qiagen, Inc., Valencia, CA). RNA was reverse transcribed using random oligodeoxynucleotide primers and the AMV First Strand cDNA synthesis kit (Roche Diagnostics Corporation, Indianapolis, IN). The resulting cDNA templates were used in qPCR amplification reactions with gene specific primer pairs (Table 1) [32]. Primers were designed using MacVector 10 software (MacVector, Inc., Cary, NC) and their target specificity was verified using NCBI-BLAST (Basic Local Alignment Search Tool). The amplified signals from triplicate reactions were detected and analyzed using the Mastercycler ep realplex instrument and software (Eppendorf AG, Hamburg, Germany). Relative mRNA abundance was calculated from the ng ratios of specific mRNA to 18S rRNA measured in the same samples. Inter-group statistical comparisons were made using the calculated mRNA/18S ratios.

**Table 1:** Primer Pairs Used for Quantitative RT-PCR Analysis

Primers*	Direction	Sequence (5'→3')	Position (mRNA)	Amplicon Size (bp)
Hu	For	CAC TGT GTG AGG GTC CAT CTT CTG	271	50
Hu	Rev	TCA AGC CAT TCC ACT CCA TCT G	320	
MAG-1	For	AAC CTT CTG TAT CAG TGC TCC TCG	18	63
MAG-1	Rev	CAG TCA ACC AAG TCT CTT CCG TG	80	
GFAP	For	TGG TAA AGA CGG TGG AGA TGC G	1245	200
GFAP	Rev	GGC ACT AAA ACA GAA GCA AGG GG	1444	
Tau	For	CGC CAG GAG TTT GAC ACA ATG	244	65
Tau	Rev	CCT TCT TGG TCT TGG AGC ATA GTG	308	
AAH	For	TGC CTG CTC GTC TTG TTT GTC	666	118
AAH	Rev	ATC CGT TCT GTA ACC CGT TGG	783	
HES-1	For	AGC GCT ACC GAT CAC AAA GT	70	143
HES-1	Rev	TCA GCT GGC ATT TTC CTT TT	212	
JAGGED-1	For	CTG AGG ACT ACG AGG GCA AG	2041	191
JAGGED-1	Rev	ACA GGT GAA TTT GCC TCC TG	2231	
NOTCH-1	For	GGT GGA CAT TGA CGA GTG TG	1918	204
NOTCH-1	Rev	CCC TTG AGG CAT AAG CAG AG	2121	
18S rRNA	For	GGA CAC GGA CAG GAT TGA CA	1278	50
18S rRNA	Rev	ACC CAC GGA ATC GAG AAA GA	1327	

Forward (For) and reverse (Rev) primer pairs used to measure gene expression by qRT-PCR. Positions of the most 5' nucleotides and the amplicon length (base pair, bp) are listed.

### Enzyme linked immunosorbent assay (ELISA)

Cerebellar homogenates were prepared in radioimmunoprecipitation assay (RIPA) buffer containing protease and phosphatase inhibitors [30,33]. Protein concentrations were determined using the bicinchoninic acid (BCA) assay (Pierce, Rockford, IL). We performed direct binding ELISAs to measure immunoreactivity. Samples containing 50 ng protein diluted in Tris buffered saline, pH 7.4 (TBS) were adsorbed to the bottom flat surfaces of 96-well polystyrene plates (Nunc, Rochester, NY) overnight at 4°C [18]. Non-specific binding sites were blocked by a 3-hour room temperature incubation with 300 μl/well of TBS + 0.05% Tween 20 + 3% BSA. Samples were then incubated with 0.1-0.5 μg/ml primary antibody for 1 h at 37°C. Immunoreactivity was detected with horseradish peroxidase (HRP)-conjugated secondary antibody and Amplex Red soluble fluorophore (Molecular Probes, Eugene, OR) [18,33]. Fluorescence was measured (Ex 530/Em 590) in a SpectraMax M5 microplate reader (Molecular Devices Corp., Sunnyvale, CA). Parallel negative control assays had primary, secondary, or both antibodies omitted. Between steps, reactions were rinsed 3 times with TBS + 0.05% Tween 20 using a Nunc ELISA plate washer.

### Sources of reagents

QuantiTect SYBR Green PCR Mix was obtained from (Qiagen Inc, Valencia, CA). Monoclonal antibodies to Notch-1, Jagged-1, β-Actin and were purchased from Abcam Inc. (Cambridge, MA). Antibodies to Hu, glial fibrillary acidic protein (GFAP), myelin-associated glycoprotein 1 (MAG-1), synaptophysin, SNAP-25, and GAP-43 were purchased from Molecular Probes (Eugene, OR), Santa Cruz Biotechnology Inc. (Santa Cruz, CA), or Chemicon International (Tecumsula, CA). The 85G6 AAH mAb was generated to human recombinant protein and purified over Protein G columns (Healthcare, Piscataway, NJ) [18].

### Statistical analysis

Data depicted in the graphs represent the means ± S.E.M.'s for each group. Inter-group comparisons were made using Student t-tests since the siAAH and siScr groups, and the pAAH and pGFP groups were studied in separate experiments. Statistical analyses were performed using the GraphPad Prism 5 software (San Diego, CA) and significant P-values (<0.05) are indicated over the graphs.

## Results

### Effects of siAAH on growth and brain weight

**Figure 1:**
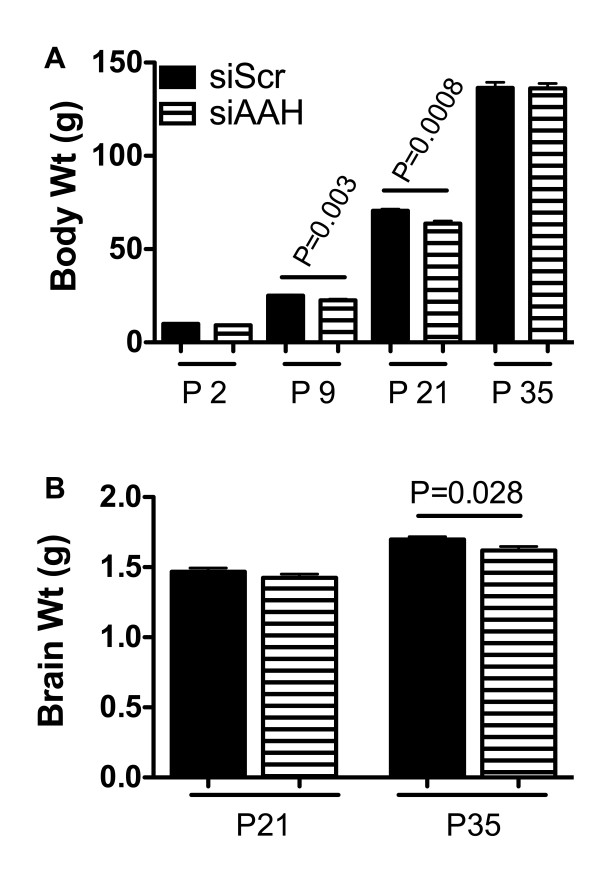
**Intracerebroventricular delivery of siAAH retards body and brain growth**. P2 Long Evans rat pups were administered intracerebro-ventricular injections of siRNA targeting AAH (siAAH) or no specific gene (siScr). Body weights were measured over the course of the experiment, and brain weights were obtained at the time of sacrifice. (A) Mean ± S.D. body weight measured on postnatal days (P) 2, 9, 21, and 35. (B) Mean ± S.D. of brain weights measured on P21 or P35. Inter-group statistical comparisons were made using Student T-test analyses. N = 8-10 rats per group. Significant P-values are provided above the graphs.

Rats were weighted on P2, P9, P21, and P35 (sacrifice) and brain weights were obtained on P21 or P35. Although initial body weights were similar for the two groups, on P9 and P21, the siAAH-treated rats had significantly lower mean body weights relative to siScr-treated controls (Figure 1A). However, by P35, the mean body weights were again similar for the two groups. On P21, the mean brain weights were similar for siAAH- and siScr-treated rats, but on P35, the siAAH-treated rats had a slight, but significantly reduced mean brain weight relative to controls (Figure 1B). In contrast, intracerebroventricular transfection with pAAH produced no significant alterations in mean brain or body weight relative to control (pGFP) (data not shown).

### Rotarod test performance

**Figure 2:**
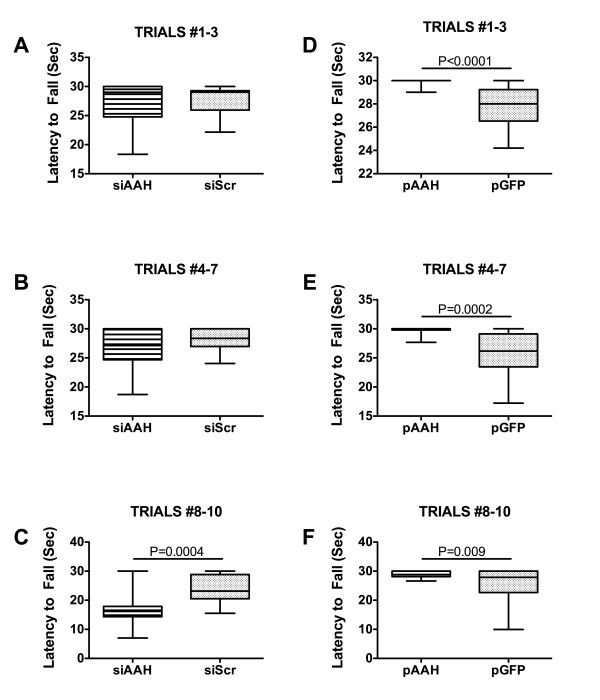
**Effects of siAAH and pAAH brain transfections on motor performance**. P2 Long Evans rat pups were administered intracerebroventricular injections of siRNA targeting AAH (siAAH) or no specific gene (siScr) (A-C), or recombinant plasmid DNA containing the full-length AAH cDNA (pAAH) or GFP (D-F). On P20, rats were subjected to 10 incremental speed trials (from 2 to 10 rpm) of rotarod testing of motor function. The maximum duration of performance was limited to 30 seconds. Data from (A, D) Trials 1-3 (2-4.5 rpm), (B, E) Trials 4-7 (5-7.5 rpm), and (C, F) Trials 8-10 (8-10 rpm) were culled and analyzed using the Mann-Whitney test. Panels display box plots with means and minimum-maximum whiskers. Significant P-values are indicated within the panels.

The Rotarod test is used to assess sensorimotor coordination and provides a highly sensitive index of damage to the cerebellum [34]. Rotarod test results were analyzed by grouping performance for Trials 1-3, 4-7, and 8-10, in which the rotation speeds were incremented from 2 to 4.5, 5 to 7.5, and 8 to 10 rpm, respectively. The mean ± S.E.M. latency to fall periods were calculated and results are depicted graphically with box plots and minimum/maximum whiskers. For the earliest (lowest speeds) set of trials, the siAAH and siScr-treated rats performed similarly (Figure 2A). For the middle set of trials, the siAAH-treated rats had a slightly shorter mean latency to fall interval, but the difference from control was not statistically significant (Figure 2B). The largest inter-group difference was observed in the final (most challenging) set of trials in which the siAAH treated rats had a significantly shorter mean latency to fall interval relative to control (Figure 2C). In contrast, for all 3 sets of trials, the pAAH-treated rats had significantly longer mean latency to fall intervals compared with the control group (Figures 2D,E,F).

### Cerebellar hypofoliation in siAAH-treated rats

**Figure 3:**
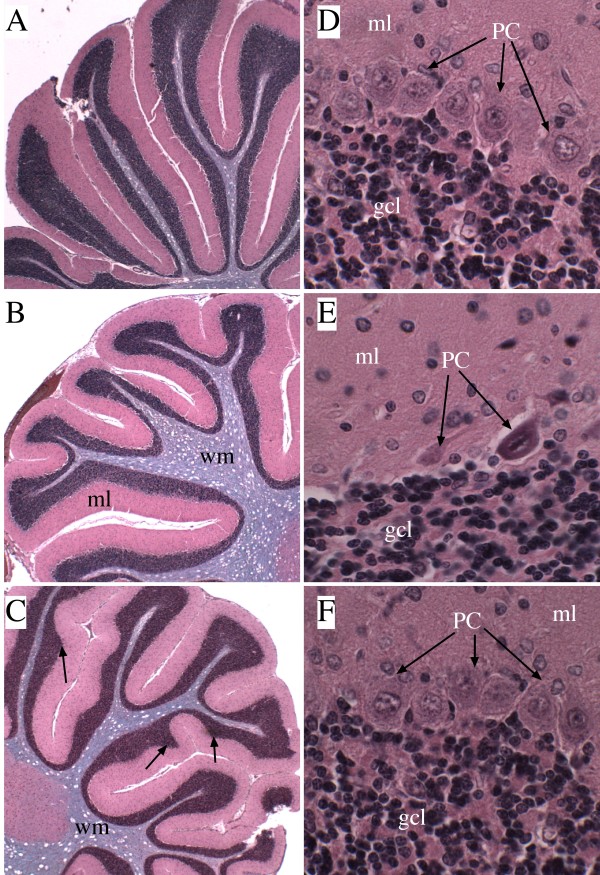
**Intracerebroventricular siAAH impairs cerebellar development**. Cerebella of rats treated by intracerebroventricular injections of siAAH, siScr, pAAH, or pGFP were harvested on P21, sectioned in the mid-sagittal plane, fixed in Histochoice, and embedded in paraffin. Histological sections were stained with LHE. (A-C) Low (100×) and (D-F) high (600×) magnification images of cerebella from (A, D) siScr, (B, E) siAAH, or (C, F) pAAH injected rats. Note long, thin, regular folia with well-developed, slender white matter (wm) cores, compact and densely populated granule (gcl) and Purkinje (PC) cell layers in cerebella of siScr- and pAAH-transfected rats, compared with the more shallow, blunted, broad, and irregular folia, thick white matter cores, irregular thickness of the granule cell layer, and neuronal loss or neuronal atrophy in the Purkinje cell layer (arrows) in cerebella of siAAH-transfected rats. (C) Cerebella of pAAH-transfected rats displayed only subtle differences from siScr or pGFP (not shown) controls in that the granule cell layer was somewhat less linear due to slightly increased architectural complexity (C; arrows). The molecular layer (ml) of the cerebella were similar in all groups.

Cerebella from P21 rats were examined histologically. Cerebella of siScr- (Figures 3A,D) and pGFP-transfected (data not shown) control rats both exhibited long, thin, regular folia with well-developed, slender white matter cores, compact and densely populated granule and Purkinje cell layers, and uniform molecular layers. In contrast, cerebella from siAAH-transfected rats had relatively more shallow, blunted, broad, and irregular folia with thick white matter cores (Figures 3B,E). In addition, the granule cell layer was highly irregular in thickness, and the Purkinje cell layer showed evidence of on-going cell loss manifested by gaps, and shrinkage and/or eosinophilia in many remaining neurons (Figure 3E). Cerebella of rats transfected with pAAH were histologically similar to control, although they did exhibit subtly increased complexity (less linear) within the granule cell layer, and greater variability in the depths of sulci (grooving) (Figures 3C,F). Otherwise, the cell densities and thickness of the cortical and white matter layers were nearly indistinguishable from control.

### Long-term effects of siAAH and pAAH on cellular gene expression in cerebella

**Figure 4:**
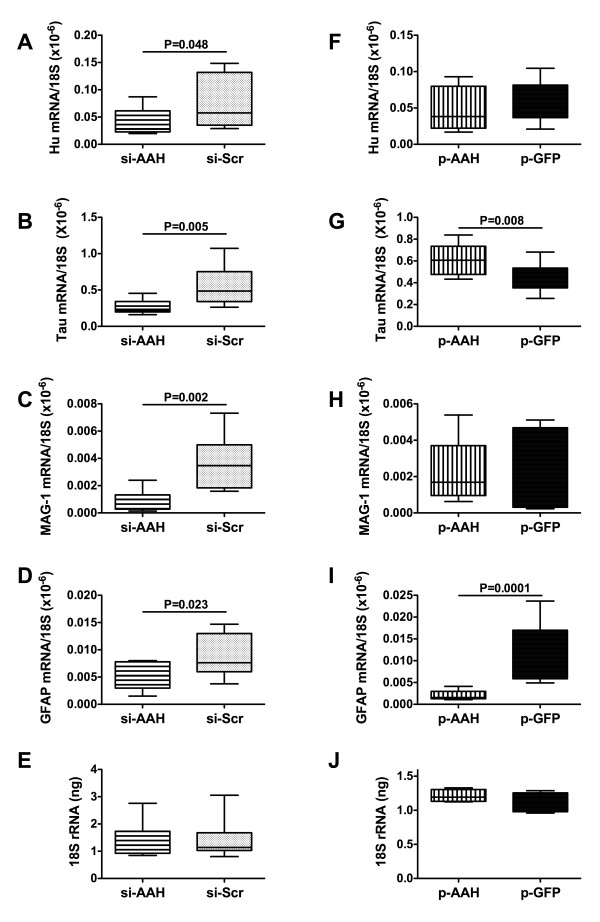
**Effects of AAH inhibition or over-expression on cell type specific gene expression**. P2 Long Evans rat pups were administered intracerebroventricular injections of (A-E) siAAH or siScr, or (F-J) recombinant plasmid pAAH or pGFP cDNA. Cerebellar tissue harvested from P21 rats was used to measure mRNA levels of (A, F) Hu, (B, G) tau, (C, H) GFAP, and (D, I) MAG-1, and (E, J) 18S rRNA by qRT-PCR. The graphs in Panels A-D and F-I depict calculated mRNA/18S ratios, and those in Panels E and J represent the mean ng rRNA input into the reactions. Panels display box plots with means and minimum-maximum whiskers. Inter-group statistical comparisons were made using Student T-tests. Significant P-values are indicated within the panels.

We used qRT-PCR analysis to examine expression of cell profile genes corresponding to neurons (Hu and tau), oligodendroglia (myelin-associated glycoprotein; MAG-1), and astrocytes (glial fibrillary acidic protein; GFAP) (Figures 4A, B, C, D, F, G, H, I). Results were normalized to 18S rRNA levels measured in the same samples (Figures 4E,J). In previous studies, we used this approach to characterize cell loss and cell type shifts associated with disease states [30,35,36]. The studies demonstrated that siAAH treatment significantly reduced the mean mRNA levels of Hu, Tau, MAG-1, and GFAP relative to control (Figures 4A. B. C, D). In contrast, the mean levels of 18S rRNA were similar in siAAH-treated and siScr control brains (Figure 4E). Brains transfected with pAAH had significantly higher mean levels of tau, and reduced expression of GFAP mRNA, but unaltered mean levels of Hu, MAG-1, and 18S relative to pGFP-transfected controls (Figures 4F, G, H, I, J).

### Effects of siAAH and pAAH on downstream notch signaling mechanisms

**Figure 5:**
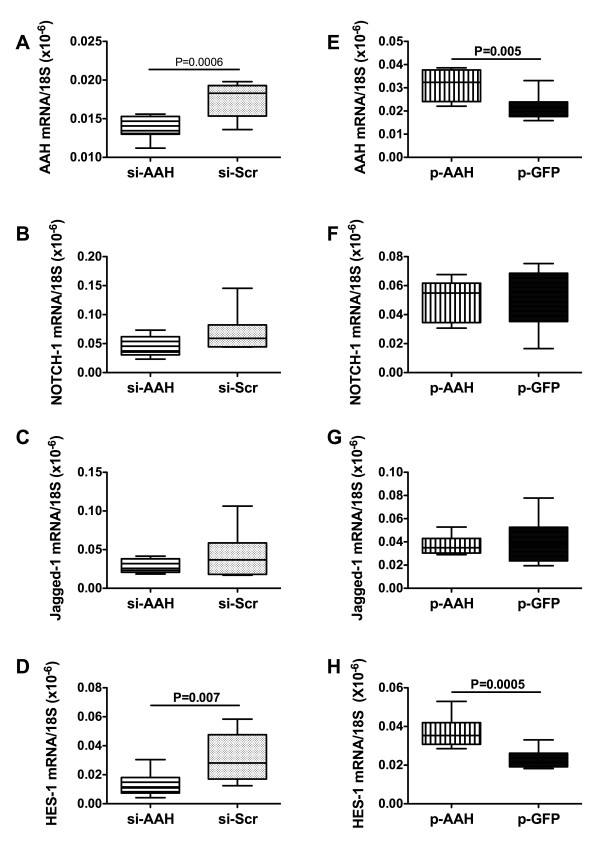
**inhibition or over-expression of AAH modulates Notch signaling mechanisms**. P2 Long Evans rat pups were administered intracerebroventricular injections of (A-D) siAAH or siScr, or (E-H) recombinant plasmid pAAH or pGFP cDNA. Cerebellar tissue harvested from P21 rats was used to measure mRNA levels of (A, E) AAH, (B, F) Notch-1, (C, G) Jagged-1, and (D, H) HES-1 by qRT-PCR, with results normalized to 18S rRNA (Figures 4E, 4J). Panels display box plots with means and minimum-maximum whiskers. Inter-group statistical comparisons were made using Student T-tests. Significant P-values are indicated within the panels.

Previous studies demonstrated that AAH protein interacts with and hydroxylates Notch and Jagged, leading to increased Notch signaling and expression of downstream target genes such as HES-1 [15]. Moreover, we previously demonstrated that intracerebral injection of young rat pups with recombinant plasmid DNA, when complexed with reagents used for in vitro transfection, can significantly and selectively increase gene expression throughout the brain and in all cell types, and with effects sustained for several weeks [29]. In both the former and current study, we monitored GFP expression, and effects of targeted gene inhibition with siRNA in brain by qRT-PCR analysis. In the present study, qRT-PCR analyses demonstrated that siRNA inhibition of AAH significantly reduced the mean cerebellar mRNA levels of AAH (Figure 5A) and HES-1 (Figure 5D), but had no significant effects on the mean mRNA levels of Notch-1 (Figure 5B) or Jagged-1 (Figure 5C) relative to siScr-transfected controls. In contrast, brains transfected with pAAH had significantly higher mean cerebellar levels of AAH (Figure 5E) and HES-1 (Figure 5H) mRNA, but similar levels of Notch-1 (Figure 5F) and Jagged-1 (Figure 5G) relative to pGFP-transfected controls.

**Figure 6:**
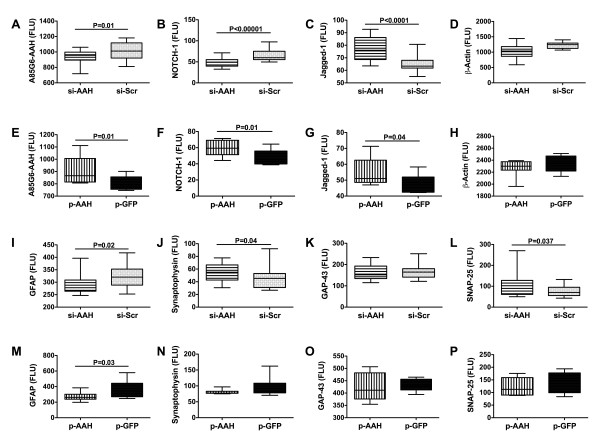
**Long term effects of siAAH and pAAH intracerebroventricular transfection on Notch signaling and plasticity proteins**. P2 Long Evans rat pups were administered intracerebroventricular injections of (A-D; I-L) siAAH or siScr, or (E-H; M-P) recombinant plasmid pAAH or pGFP cDNA. Cerebellar tissue harvested from P21 rats was used to measure immunoreactivity to (A, E) AAH-A85G6 antibody, (B, F) NOTCH-1, (C, G) Jagged-1, (D, H) β-actin, (I.M) GFAP, (J, N) synaptophysin, (K, O) GAP-43, and (L, P) SNAP-25 by direct binding ELISA. Immunoreactivity was detected with horseradish peroxidase conjugated secondary antibody and the Amplex Red fluorophore. Fluorescence was measured in a Spectramax M5 microplate reader (Ex 530 nm/Em 590 nm). Panels display box plots with means and minimum-maximum whiskers. Inter-group statistical comparisons were made using Student T-tests. Significant P-values are indicated within the panels.

ELISA studies further demonstrated that the siAAH treatments significantly reduced mean levels of AAH and Notch-1 protein, and increased Jagged-1, the ligand of Notch, but had no significant effect on the mean level of β-actin (Figures 6A, B, C, D). Transfection with pAAH significantly increased the mean levels of AAH, Notch-1 and Jagged-1 proteins, but did not significantly alter β-actin immunoreactivity relative to pGFP-transfected control cerebella (Figures 6E, F, G, H). Given the effects of siAAH and pAAH intracerebroventricular transfections on cerebellar structure and function, we entertained the hypothesis that AAH may impact synapse formation and plasticity. Therefore, we extended the analyses to measure 3 synaptic proteins, i.e. synaptophysin, which mediates synaptic communication [37], synaptosome-associated protein of 25 kD (SNAP-25), which is essential for calcium-dependent vesicle exocytosis [38], and growth-associated protein, 43 kD (GAP-43), which plays a role in neurite formation and plasticity [39]. We also measured GFAP immunoreactivity as an index of astrocyte function or activation. Those investigations demonstrated that both siAAH and pAAH transfections significantly decreased GFAP immunoreactivity in cerebella (Figures 6I,M). In addition, siAAH transfection slightly, but significantly, increased synaptophysin and SNAP-25 immunoreactivity (Figures 6J,L). In contrast, pAAH transfection had no significant effect on the mean levels of synaptophysin or SNAP-25, and neither siAAH nor pAAH significantly altered the mean levels of GAP-43 (Figure 6). Therefore, it appears that inhibition or over-expression of AAH by intracerebroventricular transfection mainly impacts cerebellar foliation and neuronal migration, while minimally influencing synaptic processes.

## Discussion

This study investigated the role of AAH in cerebellar development and function. The goal was to determine the degree to which ethanol's inhibition of AAH expression contributes to FASD-associated structural and functional abnormalities in the cerebellum. We demonstrated that intracerebroventricular transfection with siAAH significantly impairs motor function, while over-expression of AAH in the cerebellum enhances motor performance as demonstrated by rotarod testing. Importantly, although the siRNA and recombinant plasmid DNA transfections were performed on P2, the CNS effects persisted for several weeks, corresponding with results in a previous report utilizing this same experimental approach [29].

The siAAH-induced impairments in motor function were associated with conspicuous structural abnormalities in the cerebellum, including reduced foliation and decreased expression of genes that mark neurons (Hu), astrocytes (GFAP), and oligodendroglia (MAG-1). In addition, siAAH brain transfections reduced tau and GFAP expression. Together, these findings suggest that siRNA-mediated inhibition of AAH expression during postnatal cerebellar development results in net losses of neurons, oligodendroglia, and astrocytes. Since tau and GFAP represent major cytoskeletal proteins expressed in neurons and astrocytes, respectively, inhibition of AAH could promote cytoskeletal collapse and reduced inter-cellular connectivity and signaling, irrespective of relatively preserved or marginally reduced expression of synaptic plasticity proteins, including SNAP-25 and synaptophysin [37-39]. On the other hand, the significantly improved motor function and increased expression of tau associated with pAAH transfection indicate that robust AAH expression in the cerebellum during the early postnatal period could have a positive impact on subsequent cerebellar development and motor function. Therefore, ethanol's inhibition of AAH in the developing cerebellum most likely contributes to the cerebellar motor deficits in FASD.

The adverse effects of siAAH on cerebellar structure and function are highly reminiscent of previous findings in experimental models of FASD [18,25,27]. In particular, chronic gestational exposure to ethanol results in reduced cerebellar foliation with loss of neurons and oligodendrogial cells, and reduced expression of neuronal cytoskeletal proteins [18,25,27]. Moreover, ethanol exposure during development impairs motor performance due to cerebellar hypoplasia. Since AAH has a demonstrated role in mediating cell migration, which is needed for proper cerebellar foliation, reduced AAH expression in brains of siAAH-transfected rats could account for the associated perturbations in cerebellar architecture. Therefore, ethanol's inhibition of insulin/IGF stimulation of target genes, e.g. AAH, that mediate neuronal motility, contributes to some of the major CNS teratogenic effects of ethanol, including reduced cerebellar foliation and function. The adverse effects of siAAH were much less severe than those caused by early exposure to ethanol during development [18,25,27], perhaps because ethanol inhibits expression and function of many genes regulated by insulin/IGF signaling pathways, whereas siAAH targets just one of those genes and its downstream signaling through Notch [15].

Previous studies demonstrated that AAH mediates its effects on cell motility by interacting with, and hydroxylating Notch and Jagged [7], and that a downstream target of Notch signaling is the effector gene, HES-1 [40,41]. Since Notch-1 stimulates HES-1 transcription [42], the reductions in HES-1 mRNA caused by siAAH support the notion that Notch signaling is regulated by AAH. As demonstrated herein, and in previous reports, over-expression of AAH increases Notch-1 protein levels and HES-1 gene expression [15]. Previously, we showed that AAH over-expression stimulates Notch's translocation to the nucleus where it regulates gene expression [15]. Once in the nucleus, Notch-1 serves as a transcription factor for other genes involved in various functions, including motility.

Since siAAH and pAAH transfections had no significant effects on Notch's mRNA levels, AAH's regulation of Notch is likely mediated by post-translational mechanisms. For example, AAH hydroxylation of Notch leading to its translocation to the nucleus reflects post-translational regulation of Notch protein. Jagged is a ligand for Notch, and its binding to Notch is needed for Notch cleavage and release from the membrane for translocation to the nucleus [13,43,44]. The finding that pAAH increased Jagged-1 protein expression suggests an additional mechanism by which AAH regulates Notch signaling. The relevance of this observation is that Jagged and Notch are known to play critical roles in neuronogenesis and gliogenesis, and in maintaining the specialized functions of oligodendrocytes and radial glia [45-50]. Since oligodendrocytes produce central nervous system myelin and radial glia are needed for proper neuronal migration and organization of the cerebellar cortex [51,52], the impaired cerebellar foliation coupled with significantly reduced expression of Hu, MAG-1 and GFAP in siAAH-transfected brains correlate with the associated inhibition of Notch and HES-1 expression/signaling. Furthermore, siAAH may also have mediated its adverse effects on cerebellar structure by interfering with Notch signaling through sonic hedgehog [53], as sonic hedgehog mediates cerebellar foliation [54]. While all of the effects of siAAH or pAAH cannot be explained readily, conceivably some of the responses were either compensatory or regulated by yet unknown mechanisms involving pathways affected by AAH but not investigated herein.

## Conclusions

Together, these studies demonstrate a pivotal role for AAH in cerebellar development, structure, and function, and confirm that AAH expression is integrally tied to Notch-Jagged-HES-1 signaling, which regulates target genes that mediate neuronal migration and cerebellar cortical foliation in the brain. Moreover, the findings herein support the concept that ethanol inhibition of AAH expression during development mechanistically contributes to the cerebellar dysgenesis, and attendant impairments in motor function.

## List of abbreviations

AAH: Aspartyl(Asparginyl)-β-Hydroxylase; BCA: bicinchoninic acid; EGF: epidermal growth factor; FASD: fetal alcohol spectrum disorders; GFAP: glial fibrillary acidic protein; GSK-3β: glycogen synthase kinase 3β; HES-1: Hairy and Enhancer of Split 1; IGF: insulin like growth factor; IRS: insulin receptor substrate; MAG-1: myelin-associated glycoprotein 1; P: postnatal day; pAAH: recombinant plasmid DNA expressing AAH mRNA; PI3 kinase: phosphatidyl-inositol 3-kinase; qRT-PCR: quantitative Reverse Transcriptase Polymerase Chain Reaction; siAAH: siRNA targeting AAH; siRNA: small interfering RNA; SNAP-25: synaptosome-associated protein of 25 kD; TBS: Tris buffered saline, pH 7.4.

## Competing interests

The authors declare that they have no competing interests.

## Authors' contributions

ES performed the qRT-PCR and ELISA studies. PM and NB performed the neurobehavioral tests and helped analyze the data. MT generated the model, harvested the tissues, and helped with data analysis. SMD conceived of the idea, planned the experiments, supervised the research, performed statistical analysis, and generated the manuscript. All authors read and approved the final manuscript.

## Authors' information

E. Silbermann, P. Moskal, and N. Bowling are all young investigators who worked diligently to complete their first research project as pre-medical and medical students.
